# Clinical Phenotype in a Toddler with a Novel Heterozygous Mutation of the Vitamin D Receptor

**DOI:** 10.1155/2017/3905905

**Published:** 2017-05-23

**Authors:** Preneet Cheema Brar, Elena Dingle, John Pappas, Manish Raisingani

**Affiliations:** ^1^Department of Pediatrics, Division of Pediatric Endocrinology and Diabetes, New York University School of Medicine, New York, NY, USA; ^2^Department of Pediatrics, Clinical Genetics Services, New York University School of Medicine, New York, NY, USA

## Abstract

We present the clinical phenotype of a toddler who presented with vitamin D-resistant rickets, with one of the highest initial levels of alkaline phosphatase and parathyroid hormone (PTH) levels reported in the literature. The toddler had novel compound heterozygous mutations in the ligand-binding site of the vitamin D receptor and had an excellent response to calcitriol (1,25(OH)2D).

## 1. Background

Hereditary vitamin D-resistant rickets (HVDRR) is an autosomal recessive disease caused by abnormality of the vitamin D receptor (VDR). Homozygous or compound heterozygous mutations of this gene result in the inability of VDR to regulate target genes even in the abundance of 1,25-dihydroxyvitamin D (1,25(OH)2D3) which results in compensatory hyperparathyroidism with hypocalcaemia, hypophosphatemia, and osteopenia [1, 2].

Though true prevalence is not well established, there are no gender differences in the incidence, with reported cases being in consanguineous families from the Mediterranean and Middle East regions [3, 4].

There are two main functional domains in the VDR protein: DNA-binding domain (DBD) and ligand-binding domain (LBD). Mutations in the DBD prevent the VDR from binding to vitamin D response elements (VDREs) in target genes causing absolute resistance to 1,25(OH)2D resulting in a more severe clinical phenotype. Mutations in the LBD prevent binding of VDR to 1,25(OH)2D or interfere with VDR signaling [5, 6]. Alopecia is a specific clinical manifestation of HVDRR and is usually associated with DBD mutations, though it is seen in some cases. In this report, we describe clinical manifestation of the two compound heterozygous mutations in the DBD and LBD domains, respectively.

## 2. Case Report

We present a 19-month-old Hispanic female toddler with poor interval growth. Though she was meeting her developmental milestones, her height and weight were below the third percentile with weight of 8.7 kg (<3%) and height of 75 cm (2%). She was a full-term, spontaneous vaginal delivery without complications during labor or pregnancy. She was the product of nonconsanguineous marriage. She was on no medications; there were no other siblings with failure to thrive. There was no history of malabsorptive conditions.

**Figure 1:**
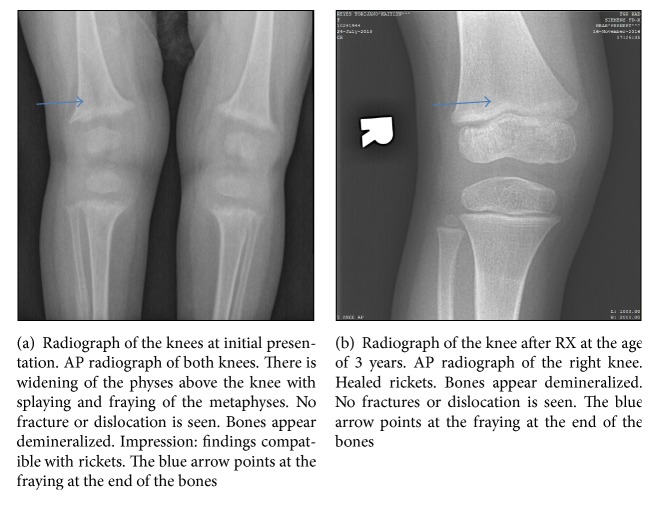


Her review of systems is negative for emesis, diarrhea, fever, appetite changes, swallowing abnormalities, respiratory symptoms, apnea, repeated acute illnesses, or frequent injuries. Her physical exam is significant for an alert, playful, developmentally appropriate child, small for her age. Her head/neck, cardiac, respiratory, gastrointestinal, genitourinary, musculoskeletal, and neurological exams were within normal limits. She had no evidence of dysmorphism and had no alopecia on exam. The laboratory testing at baseline included calcium of 7.6 mg/dl (8–10.4), alkaline phosphatase of 2023 IU/L (25–100 adult reference in our lab, with 80% bone isoenzyme), PTH of 1115 pg/ml (14–72), phosphorus of 2.9 mg/dl (2.7–4.5), 25-OH vitamin D3 of 14.2 ng/ml (30–100), and 1,25(OH)2 vitamin D3 of 505 pg/ml (19–79). The skeletal survey: Figure 1(a) shows metaphyseal fraying and cupping of the distal femur, proximal tibia, and fibula consistent with rickets.  Figure 1(b) shows healing rickets. The toddler was started on 4000 IU of ergocalciferol daily and 3 ml of calcium glubionate TID (200 mg/day; 23 mg/kg/day). 10 days later, her calcium went down to 7.3, and she received IV calcium (6 ml q6h, 64 mg/kg/day). Her calcium normalized within 48 hours and she was continued on 64 mg/kg/day of oral calcium and ergocalciferol dose of 2000 IU daily. Her calcium level was fluctuating between 7.7 and 8.3 (Table 1). At 21.5 months of age, she was started on calcitriol 0.5 mcg BID as we suspected resistant rickets. Calcitriol was gradually increased up to the current dose of 8 mcg BID. Her PTH and alkaline phosphatase were gradually trending down (Table 1). After 6 months of treatment on 8 mcg BID of calcitriol and 150–200 mg/day of elemental calcium, she had a PTH of 300 pg/ml and calcium of 8.7 mg/dl and had early radiological signs of healing rickets and clinical improvement in gait. Calcium treatment was finally stopped at 29 months of age with healing of rickets (Table 1).

## 3. Genetic Analysis

Genomic DNA was isolated from peripheral blood samples of the patient. The VDR gene was amplified by polymerase chain reaction (PCR) and all exons of the coding region of the VDR gene were directly sequenced at the Baylor Miraca Genetics Laboratories. Direct sequence analysis of PCR products was performed in both forward and reverse directions using automated fluorescence dideoxy sequencing methods. There were two compound heterozygous mutations found in this patient. One of them was a heterogenous missense mutation of Arg274 (Arg274His) in exon 9 which changed the codon for arginine to histidine at amino acid 274. The second mutation was a heterogenous missense mutation of Arg73 (Arg73Glu) in exon 5 which changed the codon for arginine to glutamine at amino acid 73. The sequence analysis also identified benign sequence variants: homozygous c.2T>C (p.M1T) polymorphism in exon 4 and c.1056T>C (p.I352I) variant in exon 11. Both parents were asymptomatic though parental analysis of the VDR sequence will be performed.

## 4. Discussion

Our toddler had a remarkable response to calcitriol, despite having one of the highest initial PTH (1115.4 pg/ml) and alkaline phosphatase (2023 IU/L) levels reported. Sequence analysis of the toddler's DNA revealed two compound heterozygous mutations in the VDR gene.

The clinical severity in case of LBD mutations depends on the affinity of VDR and varies from reduced affinity to 1,25(OH)2D3 to a total absence of binding to 1,25(OH)2D3. Amino acids 123–427 constitute the VDR LBD with R274 being the ligand-binding site located in helix H5 that makes contact with the 1*α*-hydroxyl group of 1,25(OH)2D3 [7]. Mutant R274H is similar to mutant R274L and is characterized by 100-fold less responsiveness to 1,25(OH)2D3 compared to the wild-type VDR [3, 4]. All previously reported patients with R274H and R274L were from Middle Eastern countries. Later, two case presentations of the homozygous mutant R274H were reported by Aljubeh et al. [4]. The clinical manifestation of these two patients with HVDRR was much more severe compared to our toddler. Both patients developed respiratory complications with one of them requiring oxygen supplementation. The first patient was initially started on oral calcium at the dose of 500–600 mg/kg/day with a poor response. Both of them required high doses of IV calcium infusions (90–200 mg/kg/day) for at least three months. Our toddler had a heterozygous mutation of R274H which could explain her less severe presentation, although she had 10 times higher level of PTH and two times higher level of alkaline phosphatase compared to the two cases reported by Aljubeh et al. [4]

Our case has compound heterozygosity for the Arg274His and the Arg73Glu. The residue Arg73 is located at the tip of the second zinc finger of the intracellular vitamin D receptor and it is conserved in evolution. Hughes et al. (1988) reported on two sisters from consanguineous heterozygous and asymptomatic parents of a black Haitian origin. The described mutation resulted in an Arg70Gly substitution and a decreased affinity for DNA. The reported mutation is actually Arg73Gln (R73Q) based on corrected sequencing. In the functional cDNA analysis, Hughes et al. found decreased affinity of the expressed VDR mutant to 1,25(OH)2D3. Arginine at this position is critical for the interaction of receptor with DNA. The toddler in our case report had a compound heterozygous mutation that could explain her mild symptoms of HVDRR.

The cornerstone of treatment in HDVRR is to overcome the absent or reduced affinity of the ligand 1,25(OH)2D3 for the VDR using available formulations of 1,25(OH)2D3. In the previous report by Aljubeh et al., the dose of calcitriol was 10–15 mcg/day [4] in addition to 150–600 mg/kg/day of IV elemental calcium. Our patient has sustained normal serum calcium on much higher calcitriol treatment (24 mcg/day) without the need for oral elemental calcium. As long as the urine is being monitored for hypercalciuria, normalization of the PTH is the mainstay of treatment. Healing of the rickets has been observed in some children with discontinuation of all therapy. This resolution of HDVRR may be the result of VDR-independent pathways of calcium absorption in the gut combined with effects of estrogens during puberty through upregulation of calcium transport protein 1 channels (CaT1) [8].

## 5. Conclusion

Our case illustrates compound heterozygous mutations in the VDR both in the hormone-binding and in the nuclear-binding site with a less severe presentation of rickets and a quick response to treatment with calcitriol and a short-term calcium requirement, despite having one of the highest initial PTH and alkaline phosphatase levels reported.

## Figures and Tables

**Table 1:** Metabolic parameters on diagnosis and during RX.

Time after diagnosis (weeks)	Calcium (mg/dl)	Alkaline phosphatase (IU/L)	25-OH vitamin D2 (ng/ml)	1,25(OH) vitamin D3 (pg/ml)	PTH (pg/ml)	Calcium/creatinine ratio (urine)
Baseline	7.6	2023	14	505	1115	0.15
2 weeks	7.3	1579	31	901	764	0.06
1 month	7.8	1708	20	784	924	0.31
1.5 months	7.7	1879	44	1165	866	0.29
2 months	7.9	1579	36	901	901	ND
3 months	7.9	1686	40	959	959	0.12
4 months	8.8	1343	44	2064	437	0.17
5 months	8.2	848	41	1300	536	0.3
8 months	8.7	705	33	>600	300	ND
10 months	9.5	662	36	>600	147	ND
12 months	9.5	441	58	>600	210	ND
16 months	9.4	287	45	>600	76	0.06
21 months	9.7	180	43	>600	35	0.06

Metabolic parameters (reference values): calcium = 8–10.4 mg/dl; alkaline phosphatase = 25–100 IU/L (adult reference in our lab, with 80% bone isoenzyme); PTH = 14–72 pg/ml; 25-OH vitamin D2 = 30–100 ng/ml; 1,25(OH) vitamin D3 = 19–79 pg/ml. ND: not done.
